# The Plastid Genome of the Cryptomonad *Teleaulax amphioxeia*

**DOI:** 10.1371/journal.pone.0129284

**Published:** 2015-06-05

**Authors:** Jong Im Kim, Hwan Su Yoon, Gangman Yi, Hyung Seop Kim, Wonho Yih, Woongghi Shin

**Affiliations:** 1 Department of Biology, Chungnam National University, Daejeon, Korea; 2 Department of Biological Sciences, Sungkyunkwan University, Suwon, Korea; 3 Department of Computer Science, Gangneung-Wonju National University, Wonju, Korea; 4 Department of Marine Biotechnology, Kunsan National University, Kunsan, Korea; Philipps-University Marburg, GERMANY

## Abstract

*Teleaulax amphioxeia* is a photosynthetic unicellular cryptophyte alga that is distributed throughout marine habitats worldwide. This alga is an important plastid donor to the dinoflagellate *Dinophysis caudata* through the ciliate *Mesodinium rubrum* in the marine food web. To better understand the genomic characteristics of *T*. *amphioxeia*, we have sequenced and analyzed its plastid genome. The plastid genome sequence of *T*. *amphioxeia* is similar to that of *Rhodomonas salina*, and they share significant synteny. This sequence exhibits less similarity to that of *Guillardia theta*, the representative plastid genome of photosynthetic cryptophytes. The gene content and order of the three photosynthetic cryptomonad plastid genomes studied is highly conserved. The plastid genome of *T*. *amphioxeia* is composed of 129,772 bp and includes 143 protein-coding genes, 2 rRNA operons and 30 tRNA sequences. The DNA polymerase III gene (*dna*X) was most likely acquired via lateral gene transfer (LGT) from a firmicute bacterium, identical to what occurred in *R*. *salina*. On the other hand, the *psb*N gene was independently encoded by the plastid genome without a reverse transcriptase gene as an intron. To clarify the phylogenetic relationships of the algae with red-algal derived plastids, phylogenetic analyses of 32 taxa were performed, including three previously sequenced cryptophyte plastid genomes containing 93 protein-coding genes. The stramenopiles were found to have branched out from the Chromista taxa (cryptophytes, haptophytes, and stramenopiles), while the cryptophytes and haptophytes were consistently grouped into sister relationships with high resolution.

## Introduction

The genus *Teleaulax* belongs to the class Cryptophyceae and is an ecologically important component of marine phytoplankton communities [1–7]. In addition to its importance in the trophic web, *Teleaulax* is significant in the context of kleptoplasty due to its complex relationship with the grazer ciliate *Mesodinium rubrum* and its indirect relationships with dinoflagellates, such as *Dinophysis* and *Amylax* [8–12].

Since Charles Darwin described an extensive reddish discoloration in the sea south of Valparaiso near the southern boundary of the Peru coastal current [13], the marine ciliate *Mesodinium rubrum* has been identified worldwide as a bloom-forming ciliate [12, 14–21]. *Mesodinium rubrum* contains functional chloroplasts derived from cryptophycean species of the genera *Teleaulax* and *Geminigera* [22–26]. In addition, phototrophic dinoflagellate species of the genera *Dinophysis* and *Amylax* also contain plastids of cryptophyte origin, particularly of *Teleaulax amphioxeia* and *Geminigera cryophila* origin [9, 27–34]. Cryptophyte plastids were acquired by the marine ciliate *M*. *rubrum* and then transferred to dinoflagellates via feeding. Recently, Kim et al. [9] have revealed that *Dinophysis caudata* feeds on the mixotrophic ciliate *M*. *rubrum* and secondarily retains the plastids of *T*. *amphioxeia*, transforming them into stellate compound chloroplasts. Kleptoplasty is a well-known phenomenon occurring in dinoflagellates [27, 29, 30], ciliates [24], and sacoglossan sea slugs [35, 36], which sequester chloroplasts from algal food resources. However, the longevity of a photosynthetically functional plastid in predator cytoplasm depends on the kleptoplast genome itself [37] and not on the lateral transfer of algal nuclear genes to the predator [38, 39]. Therefore, plastid genome analysis of *T*. *amphioxeia* may be important to better understand the kleptoplastic relationship between *T*. *amphioxeia* and *M*. *rubrum*.

Cryptophytes have unique secondary plastids and possess four genomes (host nuclear, mitochondrial, plastid, and nucleomorph genomes). Cryptophyte plastids display four envelope membranes, with a eukaryotic compartment between the outer and inner membrane pairs. Plastid-containing cryptophytes are important models for plastid evolution through secondary endosymbiosis between phagotrophic and photoautotrophic eukaryotes [40, 41], a process that has presumably also occurred in several other protist lineages [42, 43]. Nucleomorphs derived from red algal nuclei persist in the remnant cytosol of engulfed algal cells, between the inner and outer pairs of plastid membranes [44–46]. To date, nine cryptomonad organelle genomes, including 3 plastid, 2 mitochondrial, 3 nucleomorph and 1 nuclear genome, have been sequenced. For example, the plastid [47] and mitochondrial genomes [48] of *Rhodomonas salina* have been completely sequenced, and the nucleomorph and mitochondrial genomes of *Hemiselmis andersenii* have also been published [49, 50]. In addition, the nuclear [46], nucleomorph [51] and plastid genomes [52] of the model cryptomonad species *Guillardia theta* have been sequenced, as well as the nucleomorph [53] and plastid genomes [54] of the nonphotosynthetic cryptomonad *Cryptomonas paramecium*. More recently, the *Chroomonas mesostigmatica* nucleomorph genome was also sequenced [55].

Here, we present the complete plastid genome of *Teleaulax amphioxeia* together with analyses of its genome structure and gene content. This plastid genome sequence is the first to be reported with the full characterization of the plastid genes in the genus *Teleaulax*. Comparative analysis was conducted using the genome of *T*. *amphioxeia* and three published plastid genomes of the cryptophytes *Cryptomonas paramecium*, *Guillardia theta*, and *Rhodomonas salina*. To identify the taxonomic relationships and evolutionary history of algae with red-algal derived plastid, we reconstructed plastid phylogenies based on 93 protein-coding genes from the currently available genomic data, using 28 plastid genomes, including the genomes of 4 cryptophytes, 4 haptophytes, 12 stramenopiles, and 9 red algal species. Additionally, we demonstrate herein the conserved properties and variability of the plastid genomes among the cryptophyte lineages. Our genetic information and plastid genome comparisons among the cryptophytes provide important insights into both the evolution of organelle genomes and the harmful algae-associated trophic web in marine ecosystems.

## Materials and Methods

### DNA isolation and sequencing

A culture derived from a single-cell isolate of *Teleaulax amphioxeia* collected from Gomso Bay, Korea (35° 40’ N, 126° 40’ E), which was established in a previous study [56], was selected for genome sequencing. DNA was extracted from the cultivated sample using a QIAGEN DNEasy Blood Mini Kit (QIAGEN, Valencia, CA, USA), following the manufacturer’s instructions. A sequencing library was prepared using an Ion Xpress Plus gDNA Fragment Library Preparation Kit and an Ion OneTouch 200 Template Kit v2 DL (Life Technologies, San Francisco, CA, USA) according to the manufacturer’s protocol and sequenced with an Ion Torrent Personal Genome Machine (PGM) at the Yoon laboratory at Sungkyunkwan University (Suwon, Korea) using an Ion PGM Sequencing 200 Kit v2 (Life Technologies, San Francisco, CA, USA).

### Genome assembly and plastid contig selection

The data were trimmed (i.e., base = 80 bp, error threshold = 0.05, n ambiguities = 2) using CLC Genomics Workbench (CLC Bio, Aarhus, Denmark) prior to producing a *de novo* assembly with the default options (automatic bubble size, minimum contig length = 1,000 bp). The raw reads were then mapped to the assembled contigs (similarity = 95%, length fraction = 75%), and regions with no evidence of short-read data were removed (up to 1,000 bp). The resulting assembly had an average coverage of ~15x and included one large contig of 191,270 bp that was determined to be the plastid genome based on the following criteria: (1) BLAST searches of commonly known plastid genes against the entire assembly produced hits on this contig with significant e-values (e#10220); and (2) the genome size of 129,772 bp is consistent with the sizes of other photosynthetic cryptophyte plastid genomes, which range from 121 (*Guillardia theta* NC000926) to 136 kbp (*Rhodomonas salina* NC009573). Each contig that contained a plastid sequence was then manually aligned with Genetic Data Environment (MacGDE2.5) program [57], and a consensus sequence was produced. The assemblies were further verified by examining the paired-end distance and depth after re-mapping the reads to the assembled sequence.

### Genome annotation and sequence analysis

Databases of protein-coding genes and rRNA and tRNA genes were compiled from all previously sequenced cryptophyte plastid genomes. Preliminary annotations for the protein-coding genes were performed using GeneMarkS (http://opal.biology.gatech.edu/genemarks.cgi) to identify coding sequences (CDSs) and generate a basic option. The final annotation file was evaluated with Geneious Pro 5.1.7 (http://www.geneious.com/) using ORF Finder with the standard genetic code. After the alignments for each gene were completed, they were checked manually, and the corresponding open reading frames (ORFs) in the genome sequences were annotated. Annotations of ORFs with putative functional domains were included in the genome.

To identify the tRNA sequences, the plastid genome was submitted to tRNAscan-SE version 1.2.1 server (http://lowelab.ucsc.edu/tRNAscan-SE/). The genome was searched with the default settings, using the “Mito/Chloroplast” model. To determine the rRNA sequences, a set of known plastid rRNA sequences was extracted from the plastid genome of *Rhodomonas salina* and used as query sequences to search the *R*. *salina* genome using BLASTn. The annotated sequence was deposited into NCBI GenBank database as KP899713. The graphical gene map was designed with OrganellarGenomeDRAW program (http://ogdraw.mpimp-golm.mpg.de/).

### Gene arrangement comparisons

The three published cryptophyte plastid genomes with annotations were downloaded from GenBank [48, 52, 54]. For the structure and arrangement comparisons, the genomes were aligned using Mauve Genome Alignment version 2.2.0 [58] with the default settings. To aid in visualization, we designated the beginning of the *trn*Y and *rpl*19 markers as position 1 in each genome.

### Phylogenetic analysis

Phylogenetic analysis was conducted to determine the taxonomic relationships and evolution of the red algal-derived plastid. A dataset was created, combining 93 homologous protein-coding gene sequences in 29 plastid genomes from red algal plastid-bearing organisms, including 4 cryptophytes, 4 haptophytes, 12 stramenopiles and 9 red algae (S1 Table). The sequences of 2 chlorophytes and 1 glaucophyte were used as outgroup taxa to root the tree. The dataset was concatenated into 18,181 amino acids and a single continuous sequence of 56,565 nucleotides to initiate alignment by eye using MacGDE2.5 program (S2 Table).

Maximum likelihood (ML) phylogenetic analyses were performed using RAxML version 8.0.0 [59] with the Le and Gascuel with gamma (LG+GAMMA) model [60] for the amino acid data selected by ProtTest 3 [61] and with the general time-reversible plus gamma (GTR+GAMMA) model for the nucleotide data [62]. We used 1,000 independent tree inferences and identified the best tree with the-# option within the program. The gamma correction values and the proportion of invariable sites in the combined dataset were obtained automatically by the program (S3 Table). Bootstrap values (MLBS) were calculated using 1,000 replicates with the same substitution model.

Maximum parsimony (MP) and distance (neighbor-joining; NJ) trees were constructed from a combined dataset with PAUP* using a heuristic search algorithm with the following settings: 100 random sequence-addition replicates, tree bisection and reconnection (TBR) branch swapping, MulTrees, all characters unordered and unweighted, and branches with a maximum length of zero collapsed to yield polytomies. The MLBS for the resulting nodes were assessed using bootstrapping with 1,000 iterations on each tree. For NJ analyses, we analyzed the dataset using the Modeltest parameters (S3 Table).

The *dna*X protein encoded by the *T*. *amphioxeia* plastid was used as a query to identify and retrieve a diverse set of *dna*X, polymerase III gamma/tau and replication factor C proteins from public protein databases. The BLAST search was resulted the conserved protein domain models Cog2812 and TIGR02397. We selected 220 unambiguously aligned amino acid sequences from 90 homologous taxa. Sequences were aligned using MacGDE 2.5 and analyzed using RAxML as described above.

## Results and Discussion

### The plastid genome of *Teleaulax amphioxeia*

**Fig 1:**
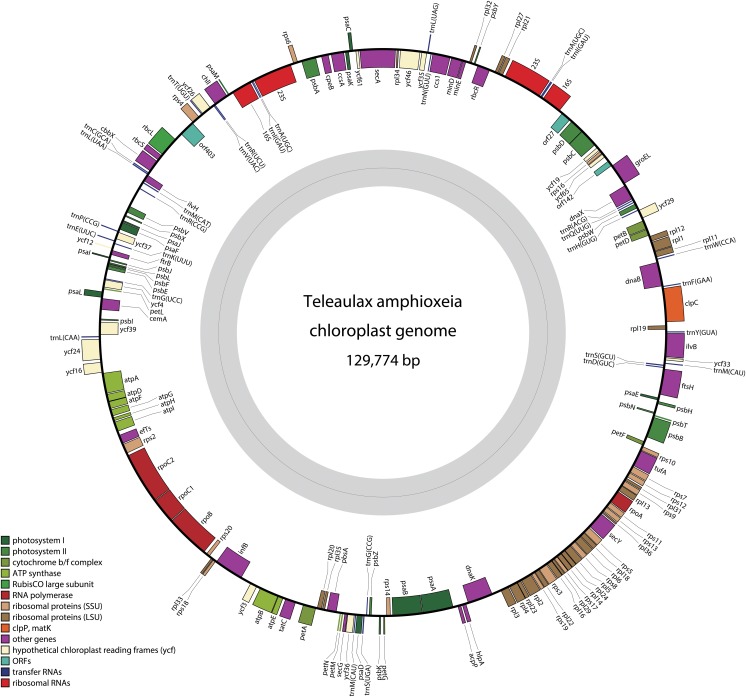
Circular map of the plastid genome of the cryptophyte *Teleaulax amphioxeia*. All of the genes are transcribed in a clockwise direction. Note the dense gene arrangement and the single large intergenic region. The protein-coding genes and ribosomal RNA and transfer RNA genes are labeled inside or outside of the circle. The genes are color coded according to the functional categories listed in the index below the map.

The plastid genome of *Teleaulax amphioxeia* was found to be 129,772 bp in size and is illustrated in Fig 1. The *T*. *amphioxeia* genome size is similar to those of *Guillardia theta* and *Rhodomonas salina*. Eighty percent of the *T*. *amphioxeia* plastid genome was predicted to consist of coding regions (Table 1), including structural RNA genes, similar to the percentages of coding regions in *G*. *theta* (87.7%), *Cryptomonas paramecium* (87.0%) and *R*. *salina* (80.8%). The proportion of intergenic space in *T*. *amphioxeia* was 15.5%, which is comparable to those of algae with red-algal derived plastid and other red algal plastid descendants (i.e., haptophytes and stramenopiles). The G+C content was 34.21% for *T*. *amphioxeia*, which is similar to those of *C*. *paramecium* (38%), *R*. *salina* (34%), and *G*. *theta* (32%). The overall G+C content was highly similar to those of other chromists and red algae [52, 63–65].

**Table 1:** Characteristics of the cryptophyte plastid genome analyzed in this study.

	*Cryptomonas paramecium* CCAP977/2a	*Rhodomonas salina* CCMP1319	*Guillardia theta*	*Teleaulax amphioxeia* HACCP CR01
**General characteristics**				
**Size (bp)**	77,717	135,854	121,524	129,772
**G+C(%)**	38%	34%	32%	34.21%
**Number of intergenic spaces (%)**	9,917 (12.8%)	23,767 (17.5%)	14,792 (12.2%)	20,108 (15.5%)
**Number of total genes (including RNAs)**	114	183	183	179
**Protein-coding genes**				
**Number of protein-coding genes**	82 (92.6%)	146 (80.8%)	147 (87.7%)	143 (80.0%)
**Number of ribosomal proteins**	42	44	44	44
**Start codon usage (no./%)**				
**ATG**	80 (97.6%)	136 (93.2%)	138 (93.9%)	135 (94.4%)
**GTG**	2 (2.4%)	10 (6.8%)	7 (4.7%)	7 (4.9%)
**TTG**	-	-	2 (1.4%)	1 (0.7%)
**Stop codon usage (no./%)**				
**TAA**	58 (70.7%)	116 (79%)	115 (78.2%)	124 (86.7%)
**TAG**	12 (14.65%)	26 (18%)	19 (12.9%)	15 (10.5%)
**TGA**	12 (14.65)	4 (3%)	13 (8.8%)	4 (2.8%)
**RNAs**				
**Number of tRNAs**	29	31	30	30
**Number of rRNA operons**	1	2	2	2
**GenBank accession**	NC013703	NC009573	NC000926	KP899713
**Pseudogene**	*atp*F			
**Missing**	*pet*, *psa*, *psb*			

The *T*. *amphioxeia* plastid genome was predicted to encode 179 genes, including 2 rRNA operons and 30 tRNA genes (Fig 1 and S4 Table). The ochre termination codon TAA was determined to be used in *T*. *amphioxeia* 86.7% of the time, and the amber (TAG) and opal (TGA) codons were found to be used 10.5% and 2.8% of the time, respectively. Seven genes contained a valine (GTG) rather than a methionine start codon (*chl*l, *hlp*A, *rpl*23, *rps*3, *rps*8, *rps*13, and *psb*C), and a TTG start codon was present in one gene (*ycf*65). A set of 136 protein-coding genes was shared by all of the plastid genomes evaluated in this study, while 170 genes were unique amongst the three photosynthetic cryptophyte species, except for *C*. *paramecium*, which is an osmotrophic, colorless species (S1 Table). An additional 107 genes, including 76 protein-coding genes were shared between *T*. *amphioxeia* and *C*. *paramecium*.

The four cryptomonads shared a similar tRNA gene set, with 30 tRNAs in *T*. *amphioxeia*, 29 tRNAs in *C*. *paramecium*, 30 tRNAs in *G*. *theta*, and 31 tRNAs in *R*. *salina* (Table 2), and their tRNAs included redundant isotypes for the amino acids 2 glycine, 2–3 serine, 3 arginine, and 3 leucine and three distinct methionine tRNAs.

**Table 2:** tRNA sequences present in the cryptophyte plastid genome.

	**trnA (GGC)**	**trnA(TGC)**	**trnC(GCA)**	**trnD(GTC)**	**trnE(TTC)**	**trnF(GAA)**	**trnG(GCC)**	**trcG(TCC)**	**trnH(GTG)**	**trnI(GAT)**	**trnK(TTT)**	**trnL(CAA)**	**trnL(GAG)**	**trnL(TAA)**	**trnL(TAG)**	**trnfM(CAT)**	**trnM(CAT)**	**trnN(GTT)**	**trnP(TGG)**	**trnQ(TTG)**	**trnR(ACG)**	**trnR(CCG)**	**trnR(CCT)**	**trnR(TCT)**	**trnS(CGA)**	**trnS(GCT)**	**trnS(GGA)**	**trnS(TGA)**	**trnT(GGT)**	**trnT(TGT)**	**trnV(GAC)**	**trnV(TAC)**	**trnW(CCA)**	**trnY(GTA)**	**Total**
*Cryptomonas paramecium*		1	1	1	1	1	1	1	1	1	1	1		1	1		3	1	1	1	1	1		1		1	1	1		1		1	1	1	29
*Rhodomonas salina*		2	1	1	1	1	1	1	1	2	1	1		1	1		3	1	1	1	1	1		2		1	1	1				1	1	1	31
*Guillardia theta*		2	1	1	1	1	1	1	1	3	1	1		1	1	1	2	1	1	1	1	1		1		1		1		1		1	1	1	30
*Teleaulax amphioxeia*		2	1	1	1	1	1	1	1	2	1	1		1	1		3	1	1	1	1	1		1		1		1		1		1	1	1	30

Similar to the two photosynthetic cryptophytes, *T*. *amphioxeia* contained two small (approximately 4.8 kb) and almost identical repeats of rRNA operons encoding 16S, 23S and 5S rRNAs and the two tRNA genes *trn*A (GAT) and *trn*I (TGC). Inverted repeats (IRs) consisting of rRNA operons (and in some cases, a few additional genes) were generally found in the plastid genomes, which represents an ancestral feature [66–68]. These repeats are present in the *G*. *theta* and *R*. *salina* genomes [47, 52], as well as in the haptophyte *Emiliania huxleyi* [65] and several diatoms [69]. *C*. *paramecium* lacks this IR arrangement, and it contains only one rRNA operon in a 16S-*trn*I-*trn*A-23S-5S configuration [54].

Four instances of overlapping genes were found in *T*. *amphioxeia*, many of which were also identified in the other chromist plastid genomes. The *psb*D-*psb*C overlap found in *T*. *amphioxeia* existed in all of the sequenced chromist genomes, although the amount of overlap varied. Overlaps involving *atp*D-*atp*F and *rpl*4-*rpl*23 were common in the stramenopiles and cryptophytes, but not in the haptophytes. Single-nucleotide overlaps between *rpl*16-*rpl*29 and *orf*142-*orf*146 were present in *T*. *amphioxeia*, similar to what was previously found in *R*. *salina* and *G*. *theta*.

### Gene content and synteny

The *T*. *amphioxeia* plastid genome was found to contain 143 predicted protein-coding genes (Table 3 and Fig 1). Overall, this genome shows a high degree of syntenic conservation with that of *R*. *salina* [47]. The gene order was generally well conserved among the four cryptomonad plastid genomes. Large tracts of complete gene order conservation were observed, such as the highly conserved and co-expressed ribosomal protein genes and the *atp* gene cluster (Fig 1).

**Table 3:** List of genes in the *Teleaulax amphioxeia* plastid genome (143 total).

Classification	Genes							
**Genetic systems**								
Maintenance	*dna*B	*rne*	*dna*X*	*min*D	*min*E	*hlp*A		
RNA polymerase	*rpo*A	*rpo*B	*rpo*C1	*rpo*C2				
Transcription factors	*rbc*R	*ycf*29						
Translation	*inf*B	*tsf*	*tuf*A					
Protein quality control	*clp*C	*dna*K	*fts*H	*gro*EL				
**Transport**								
Transport	*cem*A	*sec*A	*sec*G	*sec*Y	*suf*B (*ycf*16)	*suf*C (*ycf*24)	***tat*C**	
**ATP synthesis**								
ATP synthesis	*atp*A	*atp*B	*atp*D	*atp*E	*atp*F	*atp*G	*atp*H	*atp*I
**Ribosomal proteins**								
Large subunit	*rpl*1	*rpl*2	*rpl*3	*rpl*4	*rpl*5	*rpl*6	*rpl*11	*rpl*12
	*rpl*13	*rpl*14	*rpl*16	*rpl*18	*rpl*19	*rpl*20	*rpl*21	*rpl*22
	*rpl*23	*rpl*24	*rpl*27	*rpl*29	*rpl*31	*rpl*32	*rpl*33	*rpl*34
	*rpl*35	*rpl*36						
Small subunit	*rps*2	*rps*3	*rps*4	*rps*5	*rps*6	*rps*7	*rps*8	*rps*9
	*rps*10	*rps*11	*rps*12	*rps*13	*rps*14	*rps*16	*rps*17	*rps*18
	*rps*19	*rps*20						
**Metabolism**								
Carbohydrates	*rbc*L	*rbc*S						
Lipids	*acp*P							
Nucleotides	-							
Amino acids	*ilv*B	*ilv*H						
Cofactors	*chl*I							
**Photosystems**								
Phycobilisomes	*cpe*B							
Photosystem I	*psa*A	*psa*B	*psa*C	*psa*D	*psa*E	*psa*F	*psa*I	*psa*J
	*psa*K	*psa*L	*psa*M	*ycf*3	*ycf*4			
Photosystem II	*psb*A	*psb*B	*psb*C	*psb*D	*psb*E	*psb*F	*psb*H	*psb*I
	*psb*J	*psb*K	*psb*L	*psb*N*	*psb*T	*psb*V	*psb*W	*psb*X
	*psb*Y	*psb*Z	*ycf*12					
Cytochrome complex	*ccs*1	*ccs*A	*pet*A	*pet*B	*pet*D	*pet*F	*pet*G	*pet*L
	*pet*M	*pet*N						
Redox System	*ftr*B	*pbs*A						
**Unknown**								
Conserved ORFs	*orf*27	*orf*142	*orf*146	*orf*403	*ycf*19	*ycf*26	*ycf*33	*ycf*35
	*ycf*36	*ycf*37	*ycf*39	*ycf*46	*ycf*65			

Many proteins associated with cell and organelle division were found to be encoded within the *T*. *amphioxeia* plastid genome (Table 3) by genes including *hlp*A (encoding a chromatin-associated architectural protein), *dna*B (encoding a DNA helicase), and *min*D and *min*E (encoding proteins that prevent the creation of DNA-less “minicells” during division [70]). Specifically, *hlp*A, which encodes a histone-like protein, has been previously identified in the two photosynthetic cryptomonads. This gene is also present in the genomes of the red algae *Cyanidioschyzon merolae* [64] and *Galdieria sulphuraria* (NC024665), but it is absent from the haptophyte and stramenopile plastid genomes. The Apicomplexan *hlp*A gene was present in the nuclear genome [71–73]. Additional chaperone protein-encoding genes, such as *gro*EL and *dna*K (a member of the *hsp*70 family), were present in the *T*. *amphioxeia* plastid genome, and their products presumably participate in protein import and folding [74]. The *fts*H gene, which encodes a protease responsible for the removal of damaged D1 protein from the photosystem II (PSII) complex [37], was identified in the photosynthetic species *T*. *amphioxeia*, *R*. *salina* and *G*. *theta* but not in *C*. *paramecium*.

The components of a protein translocation system, the *sec* transport system, were maintained (*sec*A, *sec*G, and *sec*Y), and the *suf*B and *suf*C genes were also present in the *T*. *amphioxeia* plastid genome, the products of which play roles in iron-sulfur cluster assembly [75]. The identification of the *chl*I gene in the *T*. *amphioxeia* plastid genome may provide additional insights into the role of this magnesium chelatase component in plastid-to-nucleus signaling [76]. The gene encoding the *sec*-independent transport protein *tat*C was also present, as was the proteolytic degradation pathway gene *clp*C. The plastid of *T*. *amphioxeia* thus appears to have retained the ability to import necessary proteins from the cytoplasm (e.g., proteins linked to cell division) and can mediate their degradation.

The *T*. *amphioxeia* plastid genome possesses a nearly full complement of the 8 *atp* synthase subunit genes found in the other cryptomonads. These genes showed varying degrees of sequence conservation in the four cryptomonads. A total of 24 *rpl* genes encoding the 50S ribosomal subunit protein and 18 *rps* genes encoding the 30S ribosomal subunit protein were also found in *T*. *amphioxeia*. Additional genes (including *cpe*B, *ilv*B, *ilv*H, and *inf*B) were present in the plastid genome of the cryptophyte but were absent from those of the stramenopiles and/or haptophytes. The three pseudogenes (*chl*B, *chl*N, and *chl*L) encoding light-independent protochlorophyllide reductase, which is involved in the light-independent synthesis of chlorophyll [77], were identified in the *R*. *salina* plastid genome but not in the *T*. *amphioxeia* or *G*. *theta* genome (Table 3). The reverse transcriptase gene, which is present as an intron within the photosystem gene *psb*N, was identified in *R*. *salina* [47]; however, the *psb*N gene in the *T*. *amphioxeia* plastid genome lacked the reverse transcriptase gene.

### Photosynthetic genes

The gene encoding the β subunit of phycoerythrin (*cpe*B), which is part of the phycobiliprotein complex in cryptomonads, was present in the plastid genomes of *T*. *amphioxeia*, *R*. *salina* and *G*. *theta* [47, 52] but was missing from that of *C*. *paramecium* [54]. The photosynthetic regulator and electron transfer gene *ftr*B was present in all of the photosynthetic cryptophyte plastids. The *rbc*L and *rbc*S genes encoding the large and small subunits of ribulose 1, 5-bisphosphate carboxylase/oxygenase were present in all of the cryptophyte plastids, including *C*. *paramecium*.

The *psa* and *psb* gene families encode the protein subunits of photosystem I (PSI) and PSII, respectively. A total of 11 *psa* and 18 *psb* genes were present in the plastid genomes of the three photosynthetic cryptomonads [47, 52]. The loss of the *psa* and *psb* genes from the plastid genome of *C*. *paramecium* accounts for approximately 7.5 kbp of missing plastid DNA [54].

The products of the photosynthetic *pet* gene family form a complex required for oxygenic photosynthesis, particularly for noncyclic electron flow mediated by the cytochrome b6f complex [78]. The 8 *pet* genes were present in the plastid genomes of *T*. *amphioxeia*, *G*. *theta*, and *R*. *salina*; however, they were missing from that of in *C*. *paramecium*, with the curious exception of *pet*F. In other organisms that have secondarily lost their photosynthetic abilities (e.g., *Euglena longa* and *Aneura mirabilis*), the *pet* genes are either missing or have become pseudogenes [79, 80].

### Lateral gene transfer

The most unexpected finding in the *T*. *amphioxeia* and *R*. *salina* plastid genomes was the presence of a gene with strong similarity to *dna*X, which encodes the tau/gamma components of bacterial DNA polymerase [47, 81, 82]. Phylogenetic analysis revealed that the *R*. *salina* and *T*. *amphioxeia dna*X genes were acquired by lateral gene transfer (LGT), with the direct transfer of *T*. *amphioxeia dna*X from a cryptophyte alga. The *dna*X proteins of *R*. *salina* and *T*. *amphioxeia* were found to be derived from firmicutes, i.e., parasitic mycoplasmas and related organisms [47] (S1 Fig).

### Plastid genome rearrangements

**Fig 2:**
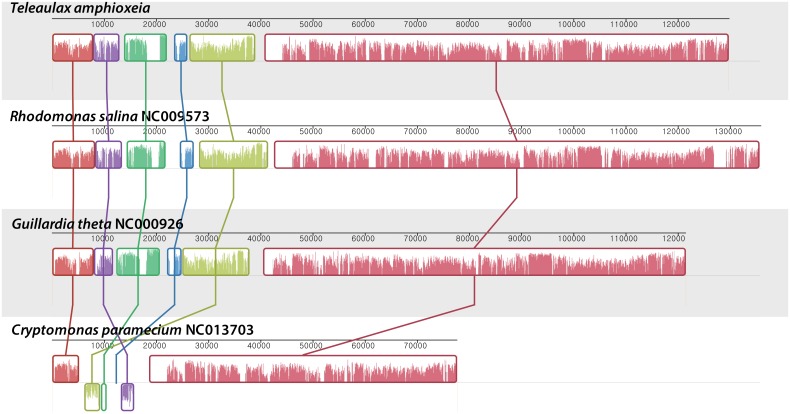
Overview of the red algal plastid genomes. Linearized maps of the complete *T*. *amphioxeia* plastid genome is compared with those of other cryptophytes. Color-coded syntenic blocks are shown above each genome, and gene maps are shown below each genome. The syntenic blocks above the horizontal line are on the same strand, and those below the line are on the opposite strand. The horizontal bars inside of the syntenic blocks indicate sequence conservation. The block boundaries correspond to sites at which inversion events occurred. On the gene maps, the genes above the horizontal line are transcribed from left to right, and those below the horizontal line are transcribed from right to left. The rRNA operons are shown in red.

Mauve pairwise alignments of the *T*. *amphioxeia* genome with each of the other four plastid genomes used in this study are shown in Fig 2. All three of the photosynthetic cryptophytes were found to have highly conserved gene arrangements and contents. All of the cryptophyte plastid genes were located in gene clusters that could be readily reconstructed from the *C*. *paramecium* genome via a small number of inversion events (Fig 2). The three photosynthetic cryptophyte plastid genomes were co-linear. *C*. *paramecium* had the smallest rearrangement distance, and almost all photosynthetic genes were found to be lost compared with the other plastid genomes; furthermore, it differed from the photosynthetic cryptophyte by only three inversions, suggesting that most of its photosynthetic genes were lost after it acquired phototrophy.

### Phylogeny

**Fig 3:**
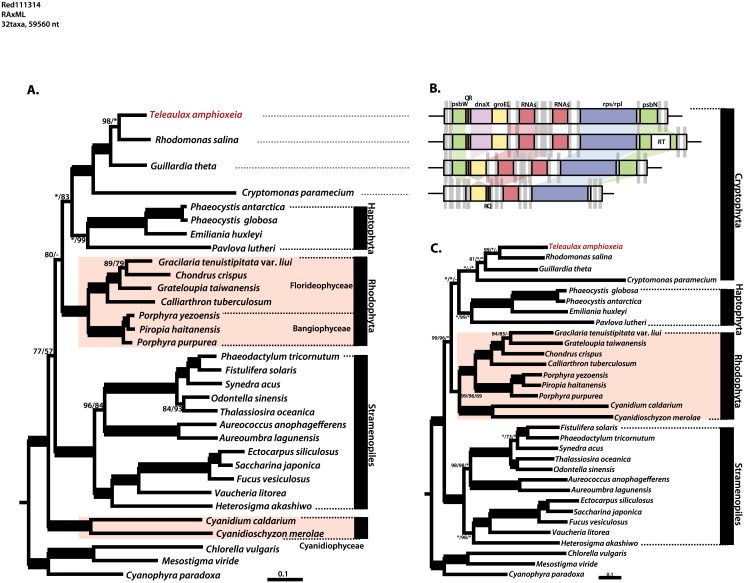
Phylogenetic tree of the cryptophyte plastids. (A) RAxML phylogeny constructed from a concatenate of 93 proteins (18,180 amino acids). The ML and MP bootstrap values are shown above or below the branches. (B) A simple overview of cryptophyte plastid gene synteny. (C) RAxML phylogeny constructed based on 93 protein-coding genes (56,569 nucleotides). The ML, MP and NJ bootstrap values are shown above or below the branches. The bold branches indicate strongly supported values (ML, MP, and NJ = 100%). The asterisks indicate supported values = 100%, and the dashes indicate values with < 50% support. The scale bar depicts the number of substitutions/site.

Phylogenomic analysis was completed using a concatenate of 93 proteins encoded by 29 complete plastid genomes from algae with red-algal derived plastid (including 4 cryptophyte, 4 haptophyte, 12 stramenopile, and 9 rhodophyte genomes) using 3 outgroups (2 chlorophyte and 1 glaucophyte genome). The sequences of chlorophyll c-containing dinoflagellates were not included due to the limited sizes of their plastid genomes. RAxML trees based on 18,180 amino acids (Fig 3A) and 56,569 nucleotides (Fig 3C) differed at the rhodophyte lineages. The cryptophyte clade was strongly supported as a monophyletic clade, which is congruent with gene synteny (Fig 3B). The resulting phylogeny suggested that the cryptophytes had sister relationships with the haptophytes in both trees. In the amino acid-based tree (Fig 3A), the Cyanidiophyceae clade was located at the base of the red-algal derived lineage, but all red algal species in the Florideophyceae and Bangiophyceae clades branched out as sisters of the Cryptophyta/Haptophyta lineage, with the exclusion of the Cyanidiophyceae clade [47, 54, 83]. In contrast with the protein ML tree, a tree based on a combined dataset of 93 protein-coding gene sequences (Fig 3C) showed that the Rhodophyta was a monophyletic clade sistered with taxa of hacrobian lineages.

Previous phylogenetic studies have demonstrated that cryptophyte, stramenopile, and haptophyte plastids are derived from a red alga [66, 84, 85]. Among the three groups (cryptophytes, haptophytes, and stramenopiles), the stramenopiles were branched outside of a cluster of chromist taxa, while the cryptophytes and haptophytes were consistently branched together as the closest relatives. These results differ from those of other analyses of five plastid genes (16S rRNA, *psa*A, *psb*A, *rbc*L, and *tuf*A), which have indicated that the stramenopiles and haptophytes are grouped together [86, 87]. The common ancestry of hacrobian (cryptophyte and haptophyte) plastids is also strongly supported ([47, 44, 83] in this study), consistent with the LGT of *rpl*36 in the cryptophyte and haptophyte plastid genomes as evidence of the sisterhood of these 2 groups and the exclusion of stramenopiles [88]. The findings of nuclear gene analyses also support this interpretation [89, 90]. However, phylogenomic data reported by other studies strongly suggest that cryptophytes and haptophytes have separate origins [91, 92]. These analyses have indicated that the haptophytes are sisters of the SAR (Stramenopile, Alveolate, and Rhizaria) group and that the cryptophytes are grouped together with the katablepharids as a broken “hacrobiana” taxa [92]. According to recent model of serial plastid endosymbioses [93], the cryptophyte plastid is more closely related to the stramenopile plastid than the haptophyte plastid. However, our phylogenies suggest the grouping together of the cryptophytes and haptophytes with moderate to high bootstrap support.

## Conclusions

We have determined the plastid genome sequence of the cryptophyte *T*. *amphioxeia*, which is the first plastid genome reported for the genus *Teleaulax*. As increasing numbers of genomes are annotated and published, comparative genomic analyses of secondary plastids will provide new insights into the patterns and processes of endosymbiosis, particularly in lineages with red-algal derived plastids. The genes that are common to all cryptophyte plastids are likely essential for plastid function and represent a useful starting point for the future annotation of plastid genomes. Several previous studies focusing on cryptophyte plastids have shown the potential of plastid genome research for answering unresolved questions about the history of these lineages, increasing our understanding of the evolution of cryptophyte plastids. The addition of the *T*. *amphioxeia* plastid genome to the suite of complete plastid genome sequences increases the breadth of plastid genomes that have been sampled to date and will help to identify common trends in organellar genomes. Many studies have shown that the *Teleaulax* species donated its plastid to the ciliate *Mesodinium rubrum* and then to the dinoflagellates *Dinophysis caudata* and *Amylax triacantha* through the trophic web and that these species have retained the acquired plastid and produce water blooms in marine ecosystems. Our *T*. *amphioxeia* plastid genome data will provide clues about the complicated plastid relationships between the donor cryptophyte *Teleaulax* and retainers, such as the ciliate *Mesodinium* and the dinoflagellates *Dinophysis* and *Amylax*.

## Supporting Information

S1 Fig The phylogenetic tree constructed from 90 *dna*X homologs. RAxML bootstrap values are shown above the branches with > 50% support. The scale bars indicate the number of substitutions/amino acid site.
(EPS)

S1 Table Cryptophyte plastid genome alignments. (XLSX)

S2 Table Concatenated data set of protein-coding genes for constructing phylogenetic tree. (XLSX)

S3 Table Evolutionary models, log likelihood values (-lnL), and model parameters proposed by ProtTest3 for amino acids [61] and Modeltest 3.7 for nucleotides [93]. (DOCX)

S4 Table The gene contents and arrangement of the *Teleaulax amphioxeia* plastid genome. (DOCX)
