# Supplementary material for: The Plastid Genome of the Cryptomonad Teleaulax amphioxeia
Source: PLoS One. 2015 Jun 5;10(6):e0129284. doi: 10.1371/journal.pone.0129284 (PMC4457928; doi:10.1371/journal.pone.0129284)
Supplement: S3 Table — (DOCX) [file pone.0129284.s004.docx]

Table S3. Evolutionary models, log likelihood values (-lnL), and model parameters proposed by the ProtTest3 for amino acids (Darriba et al. 2011) and Modeltest 3.7 for nucleotides (Posada and Crandall 1998).

| parameter/ data set | | Amino acid | |  | |  | |
| --- | --- | --- | --- | --- | --- | --- | --- |
|  | | RAxML | |  | |  | |
| Model | | LG+GAMMA+I+ | |  | |  | |
| -lnL | | 386036.1391 | |  | |  | |
| I | | 0.0636 | |  | |  | |
| G | | 0.5551 | |  | |  | |
| Tree-Length | | 6.9486 | |  | |  | |
| Amino acid frequenceies | |  | |  | |  | |
| A: 0.0791 | | Q: 0.0408 | | L: 0.0991 | | S: 0.0612 | |
| R: 0.0559 | | E: 0.0716 | | K: 0.0646 | | T: 0.0533 | |
| N: 0.0419 | | G: 0.0573 | | M: 0.0229 | | W: 0.0121 | |
| D: 0.0531 | | H: 0.0224 | | F: 0.0423 | | Y: 0.0342 | |
| C: 0.0129 | | I: 0.0622 | | P: 0.0440 | | V: 0.0692 | |
| Rate matrix | |  | |  | |  | |
| A/R: 0.3992 |  |  |  |  |  |  |  |
| A/N :0.2599 | R /N:0.7060 |  |  |  |  |  |  |
| A/D: 0.3711 | R /D:0.1164 | N/D:4.7667 |  |  |  |  |  |
| A/C: 2.3374 | R /C:0.5019 | N/C:0.4965 | D/C:0.0587 |  |  |  |  |
| A/Q: 0.9108 | R /Q:2.6367 | N/Q:1.5924 | D/Q:0.4915 | C/Q:0.0797 |  |  |  |
| A/E: 0.9752 | R /E: 0.3418 | N/E:0.5087 | D/E:4.9242 | C/E:0.0033 | Q/E: 3.8769 |  |  |
| A/G: 1.9401 | R /G:0.3664 | N/G:1.3500 | D/G:0.7934 | C/G:0.5346 | Q/G: 0.2516 | E/G: 0.3276 |  |
| A/H: 0.3369 | R /H:2.2787 | N/H:4.2344 | D/H:0.8706 | C/H:0.6015 | Q/H: 4.5201 | E/H: 0.3980 | G/H:0.2925 |
| A/I: 0.1407 | R /I: 0.1193 | N/I:0.1798 | D/I: 0.0101 | C/I: 0.3011 | Q/I: 0.0684 | E/I: 0.0416 | G/I:0.0082 |
| A/L: 0.3712 | R /L: 0.2834 | N/L:0.0643 | D/L:0.0142 | C/L:0.5578 | Q/L: 0.5469 | E/L: 0.0654 | G/L:0.0416 |
| A/K: 0.5038 | R /K:5.9405 | N/K:2.0143 | D/K:0.2657 | C/K:0.0125 | Q/K: 3.0372 | E/K: 1.6970 | G/K:0.2786 |
| A/M: 1.0555 | R /M:0.4546 | N/M:0.3484 | D/M:0.0239 | C/M:0.8392 | Q/M: 1.5706 | E/M: 0.1631 | G/M:0.1310 |
| A/F: 0.2382 | R /F: 0.0495 | N/F:0.0841 | D/F:0.0164 | C/F:1.0379 | Q/F: 0.0337 | E/F: 0.0177 | G/F:0.0841 |
| A/P: 1.1059 | R /P: 0.3123 | N/P:0.1519 | D/P:0.3704 | C/P:0.0708 | Q/P: 0.5862 | E/P: 0.3938 | G/P:0.1849 |
| A/S: 4.4391 | R /S: 0.8058 | N/S:3.7640 | D/S:1.1647 | C/S:2.6148 | Q/S: 1.1492 | E/S: 0.5747 | G/S:1.6339 |
| A/T: 2.0091 | R /T: 0.5437 | N/T:1.8787 | D/T:0.3999 | C/T:1.0738 | Q/T: 1.0143 | E/T: 0.5677 | G/T:0.1219 |
| A/W:0.1697 | R /W:0.5574 | N/W:0.0426 | D/W:0.0281 | C/W:0.6293 | Q/W:0.2218 | E/W: 0.0731 | G/W:0.2521 |
| A/Y: 0.2056 | R /Y: 0.2953 | N/Y:0.5747 | D/Y:0.1269 | C/Y:1.0945 | Q/Y: 0.2417 | E/Y: 0.1127 | G/Y:0.0513 |
| A/V: 2.3926 | R /V: 0.1605 | N/V:0.0786 | D/V:0.0357 | C/V:1.8399 | Q/V: 0.1975 | E/V: 0.2301 | G/V:0.0720 |
| G/H:0.2925 |  |  |  |  |  |  |  |
| G/I: 0.0082 | H/I: 0.1022 |  |  |  |  |  |  |
| G/L:0.0416 | H/L:0.3439 | I/L: 3.8924 |  |  |  |  |  |
| G/K:0.2786 | H/K:0.6548 | I/K: 0.1494 | L/K:0.1291 |  |  |  |  |
| G/M:0.1311 | H/M:0.4155 | I/M:4.0131 | L/M:5.9276 | K/M:0.6166 |  |  |  |
| G/F:0.0841 | H/F:0.6406 | I/F: 1.0449 | L/F: 2.4347 | K/F:0.0225 | M/F:1.6892 |  |  |
| G/P:0.1849 | H/P:0.4778 | I/P: 0.0735 | L/P: 0.2339 | K/P:0.3665 | M/P:0.0938 | F/P:0.0887 |  |
| G/S:1.6339 | H/S:0.9297 | I/S: 0.0602 | L/S: 0.1712 | K/S:0.7030 | M/S:0.3258 | F/S:0.3398 | P/S:1.2566 |
| G/T:0.1219 | H/T:0.5486 | I/T: 0.9707 | L/T: 0.2845 | K/T:1.0676 | M/T:1.8972 | F/T:0.1549 | P/T:0.5366 |
| G/W:0.2521 | H/W:0.5607 | I/W:0.1049 | L/W:0.5819 | K/W:0.0469 | M/W:0.6537 | F/W:2.3073 | P/W:0.0893 |
| G/Y:0.0513 | H/Y:4.9834 | I/Y: 0.2184 | L/Y:0.2814 | K/Y:0.1239 | M/Y:0.4519 | F/Y:7.3282 | P/Y:0.0842 |
| G/V:0.0720 | H/V:0.1118 | I/V: 10.000 | L/V:1.5989 | K/V:0.1739 | M/V:1.7829 | F/V:0.6148 | P/V:0.2784 |
| S/T:6.0778 |  |  |  |  |  |  |  |
| S/W:0.2337 | T/W:0.1322 |  |  |  |  |  |  |
| S/Y:0.3761 | T/Y:0.2309 | W/Y:0.9597 |  |  |  |  |  |
| S/V:0.0924 | T/V:2.0548 | W/V:0.1779 | Y/V:0.2341 |  |  |  |  |
| parameter/ data set | | Nucleotides | |  | |  | |
|  | | RAxML | |  | |  | |
| Model | | GTR+GAMMA+I | |  | |  | |
| -lnL | | 1042200.3579 | |  | |  | |
| I | | 0.2408 | |  | |  | |
| G | | 0.9947 | |  | |  | |
| Base frequencies | |  | |  | |  | |
| A: 0.3336 | | C: 0.1545 | | G: 0.1972 | | T: 0.3147 | |
| Rate matrix (G/T=1.0) | |  | |  | |  | |
| A/C: 4.1358 | | A/G: 5.2328 | | A/T: 3.4047 | | C/G: 3.1535 | |
| C/T: 9.1280 | |  | |  | |  | |
