# Supplementary material for: The Plastid Genome of the Cryptomonad Teleaulax amphioxeia
Source: PLoS One. 2015 Jun 5;10(6):e0129284. doi: 10.1371/journal.pone.0129284 (PMC4457928; doi:10.1371/journal.pone.0129284)
Supplement: S4 Table — (DOCX) [file pone.0129284.s005.docx]

|  | **Table S4. The gene contents and coordination of *Teleaulax amphioxeia* HACCP CR01 plastid genome.** | | | | | | | |
| --- | --- | --- | --- | --- | --- | --- | --- | --- |
|  | **start position** | **end positon** | **direction (+/-)** | **gene name** | **Gene product** | **Length** | **Start Codon** | **Stop Codon** |
| **CDS** | 213 | 581 | - | rpl19 | ribosomal protein L19 | 369 | ATG | TAA |
|  | 771 | 3230 | + | clpC | Clp protease ATP binding subunit | 2460 | ATG | TAA |
|  | 3405 | 5189 | - | dnaB | DNA replication helicase | 1785 | ATG | TAA |
|  | 5552 | 5977 | + | rpl11 | ribosomal protein L11 | 426 | ATG | TAA |
|  | 6000 | 6704 | + | rpl1 | ribosomal protein L1 | 705 | ATG | TAA |
|  | 6760 | 7149 | + | rpl12 | ribosomal protein L12 | 390 | ATG | TAA |
|  | 7226 | 7708 | - | petD | cytochrome b6/f complex subunit IV | 483 | ATG | TAA |
|  | 7741 | 8388 | - | petB | cytochrome b6 | 648 | ATG | TAA |
|  | 8571 | 9206 | + | ycf29 | TctD-like protein | 636 | ATG | TAA |
|  | 9397 | 9747 | - | psbW | photosystem II protein W | 351 | ATG | TAG |
|  | 10148 | 11281 | - | dnaX | DNA polymerase III, gamma/tau | 1134 | ATG | TAA |
|  | 11406 | 12995 | + | groEL | chaperonin GroEL | 1590 | ATG | TAA |
|  | 13011 | 13442 | - | orf142 | ORF142 | 432 | ATG | TAA |
|  | 13812 | 14110 | - | ycf65 | putative ribosomal protein 3 | 300 | TTG | TAG |
|  | 14133 | 14369 | - | rps16 | ribosomal protein S16 | 237 | ATG | TAA |
|  | 14442 | 14720 | - | ycf19 | hypothetical plastid protein 19 | 279 | ATG | TAG |
|  | 14956 | 16341 | - | psbC | photosystem II 44 kDa protein | 1386 | GTG | TAA |
|  | 16325 | 17380 | - | psbD | photosystem II protein D2 | 1056 | ATG | TAA |
|  | 17545 | 18288 | - | orf27 | Ycf27 | 744 | ATG | TAA |
| -2 | 23448 | 23777 | + | rpl21 | ribosomal protein L21 | 330 | ATG | TAA |
| -2 | 23812 | 24069 | + | rpl27 | ribosomal protein L27 | 258 | ATG | TAA |
| -2 | 24460 | 25398 | - | rbcR | LysR transcriptional regulator | 939 | ATG | TAA |
| -2 | 25599 | 25712 | + | psbY | photosystem II protein Y | 114 | ATG | TAA |
| -2 | 25886 | 26065 | + | rpl32 | ribosomal protein L32 | 180 | ATG | TAA |
| -2 | 26286 | 26561 | - | minE | septum-site determining protein, prevent the creation of DNA-less "mini-cells" during divisions | 276 | ATG | TAA |
| -2 | 26585 | 27394 | - | minD | septum-site determining protein, prevent the creation of DNA-less "mini-cells" during divisions | 810 | ATG | TAA |
| -2 | 27484 | 28797 | - | ccs1 | c-type cytochrome biogenensis protein | 1314 | ATG | TAA |
| -2 | 29220 | 29615 | - | ycf35 | Ycf35 | 396 | ATG | TAA |
|  | 29715 | 31187 | - | ycf46 | Ycf46 | 1473 | ATG | TAA |
|  | 31295 | 31435 | - | rpl34 | ribosomal protein L34 | 141 | ATG | TAA |
|  | 31518 | 34148 | - | secA | preprotein translocase subunit SecA | 2631 | ATG | TAA |
|  | 34218 | 34448 | - | ycf61 | DNA-directed RNA polymerase subunit omega | 231 | ATG | TAA |
|  | 34633 | 34878 | + | psaC | photosystem I subunit VII | 246 | ATG | TAA |
|  | 24952 | 35215 | - | psaK | photosystem I subunit X | 264 | ATG | TAA |
|  | 35313 | 36263 | - | ccsA | cytochrome c biogenesis protein | 951 | ATG | TAA |
|  | 36456 | 36989 | - | cpeB | phycoerythrin beta subunit | 534 | ATG | TAA |
|  | 37396 | 38478 | - | psbA | photosystem II protein D1 | 1083 | ATG | TAA |
|  | 38750 | 39079 | + | rps6 | ribosomal protein S6 | 330 | ATG | TAA |
|  | 44285 | 44377 | + | psaM | photosystem I subunit XII | 93 | ATG | TAA |
|  | 44483 | 45547 | + | chlI | Mg-protoporyphyrin IX chelatase | 1065 | GTG | TAA |
|  | 45965 | 46699 | + | ycf26 | two-component sensor kinase | 735 | ATG | TGA |
|  | 47032 | 47640 | + | rps4 | ribosomal protein S4 | 609 | ATG | TAA |
|  | 47688 | 48851 | - | orf403 | ORF403 | 1164 | ATG | TAA |
|  | 49414 | 50880 | + | rbcL | ribulose-1 | 1467 | ATG | TAA |
|  | 50965 | 51384 | + | rbcS | ribulose-1 | 420 | ATG | TAA |
|  | 51487 | 52371 | + | cbbX | CbbX, Rubisco expresion Protein | 885 | ATG | TAG |
|  | 52706 | 53212 | - | ilvH | acetohydroxyacid synthase small subunit | 507 | ATG | TAA |
|  | 53939 | 55199 | - | rne | ribonuclease E | 1260 | ATG | TAA |
|  | 55335 | 55820 | - | psbV | photosystem II cytochrome c550 | 486 | ATG | TAA |
|  | 56043 | 56162 | - | psbX | photosystem II protein X | 120 | ATG | TAG |
|  | 56459 | 56587 | - | psaJ | photosystem I subunit IX | 129 | ATG | TAA |
|  | 56613 | 57179 | - | psaF | photosystem I subunit III | 567 | ATG | TAG |
|  | 57393 | 57923 | - | ycf37 | conserved hypothetical protein 37 | 531 | ATG | TGA |
|  | 58620 | 58724 | + | ycf12 | Ycf12 | 105 | ATG | TAA |
|  | 58790 | 59131 | - | ftrB | ferredoxin thioreductase subunit beta | 342 | ATG | TAA |
|  | 59322 | 59432 | + | psaI | photosystem I subunit VIII | 111 | ATG | TAA |
|  | 59487 | 59606 | - | psbJ | photosystem II protein J | 120 | ATG | TAA |
|  | 59700 | 59816 | - | psbL | photosystem II protein L | 117 | ATG | TAA |
|  | 59826 | 59954 | - | psbF | photosystem II protein VI | 129 | ATG | TAG |
|  | 59973 | 60227 | - | psbE | photosystem II protein V | 255 | ATG | TAA |
|  | 61001 | 61567 | - | ycf4 | photosystem I assembly protein Ycf4 | 567 | ATG | TGA |
|  | 61641 | 61736 | - | petL | cytochrome b6/f complex subunit VI | 96 | ATG | TAA |
|  | 61898 | 62332 | + | psaL | photosystem I subunit XI | 435 | ATG | TAA |
|  | 62382 | 63218 | - | cemA | envelope membrane protein | 837 | ATG | TAA |
|  | 63875 | 63991 | - | psbI | photosystem II protein I | 117 | ATG | TAA |
|  | 64115 | 65059 | - | ycf39 | Ycf39 | 945 | ATG | TAA |
|  | 65364 | 66848 | + | ycf24 | putative ABC transporter | 1485 | ATG | TAA |
|  | 67044 | 67799 | + | ycf16 | sulfate ABC transporter protein | 756 | ATG | TAA |
|  | 67859 | 69367 | - | atpA | ATP synthase CF1 alpha subunit | 1509 | ATG | TAG |
|  | 69423 | 69974 | - | atpD | ATP synthase CF1 delta subunit | 552 | ATG | TAA |
|  | 69974 | 70513 | - | atpF | ATP synthase CF0 B subunit | 540 | ATG | TAA |
|  | 70581 | 71075 | - | atpG | ATP synthase CF0 B' subunit | 495 | ATG | TAA |
|  | 71193 | 71441 | - | atpH | ATP synthase CF0 C subunit | 249 | ATG | TAA |
|  | 71518 | 72258 | - | atpI | ATP synthase CF0 A subunit | 741 | ATG | TAA |
|  | 72542 | 73201 | - | etsf | elongation factor Ts | 660 | ATG | TAA |
|  | 73260 | 73982 | - | rps2 | ribosomal protein S2 | 723 | ATG | TAA |
|  | 74157 | 77960 | - | rpoC2 | RNA polymerase beta'' subunit | 3804 | ATG | TAA |
|  | 77991 | 79862 | - | rpoC1 | RNA polymerase beta' subunit | 1872 | ATG | TAA |
|  | 79899 | 83195 | - | rpoB | RNA polymerase beta subunit | 3297 | ATG | TAA |
|  | 83407 | 83688 | - | rps20 | ribosomal protein S20 | 282 | ATG | TAA |
|  | 83883 | 84053 | + | rpl33 | ribosomal protein L33 | 171 | ATG | TAA |
|  | 84070 | 84291 | + | rps18 | ribosomal protein S18 | 222 | ATG | TAA |
|  | 84335 | 86497 | - | infB | translation initiation factor 2 | 2163 | ATG | TAA |
|  | 87284 | 87805 | + | ycf3 | photosystem I assembly protein Ycf3 | 522 | ATG | TAA |
|  | 88074 | 89498 | + | atpB | ATP synthase CF1 beta subunit | 1425 | ATG | TAA |
|  | 89508 | 89900 | + | atpE | ATP synthase CF1 epsilon subunit | 393 | ATG | TAA |
|  | 90078 | 90863 | + | tatC | Sec-independent translocase component C | 786 | ATG | TAA |
|  | 91439 | 92392 | + | petA | cytochrome f | 954 | ATG | TAA |
|  | 92470 | 92811 | - | rpl20 | ribosomal protein L20 | 342 | ATG | TAA |
|  | 92854 | 93051 | - | rpl35 | ribosomal protein L35 | 198 | ATG | TAA |
|  | 93205 | 93915 | - | pbsA | heme oxygenase | 711 | ATG | TAA |
|  | 94169 | 94258 | + | petN | cytochrome b6/f complex subunit VIII | 90 | ATG | TAA |
|  | 94323 | 94421 | + | petM | cytochrome b6-f complex subunit VII | 99 | ATG | TAA |
|  | 94524 | 94739 | + | secG | preprotein translocase SecG subunit | 216 | ATG | TAG |
|  | 94751 | 95248 | + | ycf36 | hypothetical plastid protein 36 | 498 | ATG | TGA |
|  | 95421 | 95846 | + | psaD | photosystem I subunit II | 426 | ATG | TAA |
| # | 96301 | 96489 | - | psbZ | photosystem II protein Z | 189 | ATG | TAA |
|  | 97039 | 97176 | + | psbK | photosystem II protein K | 138 | ATG | TAA |
|  | 97362 | 97475 | + | petG | cytochrome b6/f complex subunit V | 114 | ATG | TAA |
|  | 97581 | 97883 | - | rps14 | ribosomal protein S14 | 303 | ATG | TAA |
|  | 98023 | 100227 | - | psaB | photosystem I P700 chlorophyll a apoprotein A2 | 2205 | ATG | TAA |
|  | 100254 | 102512 | - | psaA | photosystem I P700 chlorophyll a apoprotein A1 | 2259 | ATG | TAA |
|  | 102835 | 103080 | + | acpP | acyl carrier protein | 246 | ATG | TAG |
|  | 103177 | 103467 | + | hlpA | DNA binding protein hu-like protein, histon like protein | 291 | GTG | TAA |
|  | 103632 | 105515 | - | dnaK | heat shock protein 70 | 1884 | ATG | TAA |
|  | 106185 | 106799 | + | rpl3 | ribosomal protein L3 | 615 | ATG | TAG |
|  | 106855 | 107505 | + | rpl4 | ribosomal protein L4 | 651 | ATG | TAA |
|  | 107498 | 107800 | + | rpl23 | ribosomal protein L23 | 303 | GTG | TAA |
|  | 107842 | 108669 | + | rpl2 | ribosomal protein L2 | 828 | ATG | TAA |
|  | 108703 | 108987 | + | rps19 | ribosomal protein S19 | 285 | ATG | TAA |
|  | 109004 | 109351 | + | rpl22 | ribosomal protein L22 | 348 | ATG | TAA |
|  | 109371 | 110033 | + | rps3 | ribosomal protein S3 | 663 | GTG | TAA |
|  | 110071 | 110484 | + | rpl16 | ribosomal protein L16 | 414 | ATG | TAA |
|  | 110488 | 110694 | + | rpl29 | ribosomal protein L29 | 207 | ATG | TAA |
|  | 110712 | 110984 | + | rps17 | ribosomal protein S17 | 273 | ATG | TAA |
|  | 110988 | 111353 | + | rpl14 | ribosomal protein L14 | 366 | ATG | TAA |
|  | 111353 | 111688 | + | rpl24 | ribosomal protein L24 | 336 | ATG | TAA |
|  | 111693 | 112235 | + | rpl5 | ribosomal protein L5 | 543 | ATG | TAA |
|  | 112267 | 112665 | + | rps8 | ribosomal protein S8 | 399 | GTG | TAA |
|  | 112689 | 113228 | + | rpl6 | ribosomal protein L6 | 540 | ATG | TAA |
|  | 113256 | 113612 | + | rpl18 | ribosomal protein L18 | 357 | ATG | TAA |
|  | 113631 | 114137 | + | rps5 | ribosomal protein S5 | 507 | ATG | TAA |
|  | 114210 | 115472 | + | secY | preprotein translocase subunit SecY | 1263 | ATG | TAG |
|  | 115537 | 115683 | + | rpl36 | ribosomal protein L36 | 147 | ATG | TAG |
|  | 115726 | 116094 | + | rps13 | ribosomal protein S13 | 369 | GTG | TAA |
|  | 116129 | 116521 | + | rps11 | ribosomal protein S11 | 393 | ATG | TAA |
|  | 116565 | 117509 | + | rpoA | RNA polymerase alpha subunit | 945 | ATG | TAA |
|  | 117570 | 118001 | + | rpl13 | ribosomal protein L13 | 432 | ATG | TAA |
|  | 118014 | 118412 | + | rps9 | ribosomal protein S9 | 399 | ATG | TAA |
|  | 118446 | 118655 | + | rpl31 | ribosomal protein L31 | 210 | ATG | TAA |
|  | 118742 | 119116 | + | rps12 | ribosomal protein S12 | 375 | ATG | TAA |
|  | 119152 | 119622 | + | rps7 | ribosomal protein S7 | 471 | ATG | TAA |
|  | 119687 | 120913 | + | tufA | elongation factor Tu | 1227 | ATG | TAA |
|  | 120939 | 121247 | + | rps10 | ribosomal protein S10 | 309 | ATG | TAG |
|  | 121326 | 121619 | - | petF | ferredoxin | 294 | ATG | TAA |
|  | 121995 | 123524 | + | psbB | photosystem II 47 kDa protein | 1530 | ATG | TAA |
|  | 123594 | 123692 | + | psbT | photosystem II protein T | 99 | ATG | TAA |
|  | 123759 | 123890 | - | psbN | photosystem II protein N | 132 | ATG | TAA |
|  | 124522 | 124725 | + | psbH | photosystem II protein H | 204 | ATG | TAA |
|  | 124793 | 124981 | - | psaE | photosystem I subunit IV | 189 | ATG | TAA |
|  | 125233 | 127119 | + | ftsH | cell division protein | 1887 | ATG | TAA |
|  | 127759 | 127956 | + | ycf33 | hypothetical plastid protein 33 | 198 | ATG | TAA |
|  | 128004 | 129728 | + | ilvB | acetohydroxyacid synthase large subunit | 1725 | ATG | TAG |
|  |  |  |  |  |  |  |  |  |
| RNAs | 5 | 86 | + | trnY(GUA) |  | 82 |  |  |
|  | 3327 | 3399 | + | trnF(GAA) |  | 73 |  |  |
|  | 5360 | 5432 | + | trnW(CCA) |  | 73 |  |  |
|  | 9198 | 9270 | - | trnH(GUG) |  | 73 |  |  |
|  | 9914 | 9843 | - | trnQ(UUG) |  | 72 |  |  |
|  | 10077 | 10004 | - | trnR(ACG) |  | 74 |  |  |
|  | 18557 | 20045 | + | 16S |  | 1490 |  |  |
|  | 20144 | 20217 | + | trnI(GAU) |  | 74 |  |  |
|  | 20221 | 20293 | + | trnA(UGC) |  | 73 |  |  |
|  | 20363 | 23264 | + | 23S |  | 2902 |  |  |
|  | 23277 | 23397 | + | 5S |  | 121 |  |  |
|  | 28946 | 28875 | - | trnN(GUU) |  | 72 |  |  |
|  | 29125 | 29207 | + | trnL(UAG) |  | 83 |  |  |
|  | 39174 | 39291 | - | 5S |  | 121 |  |  |
|  | 39312 | 42213 | - | 23S |  | 2902 |  |  |
|  | 42283 | 42355 | - | trnA(UGC) |  | 73 |  |  |
|  | 42359 | 42432 | - | trnI(GAU) |  | 74 |  |  |
|  | 42527 | 44014 | - | 16S |  | 1490 |  |  |
|  | 45553 | 45625 | - | trnR(UCU) |  | 73 |  |  |
|  | 45645 | 45716 | - | trnV(UAC) |  | 72 |  |  |
|  | 45728 | 46800 | + | trnT(UGU) |  | 73 |  |  |
|  | 52515 | 52585 | + | trnC(GCA) |  | 71 |  |  |
|  | 52615 | 52697 | + | trnL(UAA) |  | 83 |  |  |
|  | 53311 | 53396 | - | trnM(CAT) |  | 86 |  |  |
|  | 53814 | 53886 | - | trnR(CCG) |  | 73 |  |  |
|  | 57328 | 57401 | + | trnP(UGG) |  | 74 |  |  |
|  | 58068 | 58140 | + | trnE(UUC) |  | 73 |  |  |
|  | 58241 | 58312 | - | trnK(UUU) |  | 72 |  |  |
|  | 60908 | 60978 | - | trnG(UCC) |  | 71 |  |  |
|  | 65147 | 65228 | + | trnL(CAA) |  | 82 |  |  |
|  | 95293 | 95366 | + | trnM(CAT) |  | 74 |  |  |
|  | 95898 | 95985 | + | trnS(UGA) |  | 88 |  |  |
|  | 96093 | 96164 | - | trnG(GCC) |  | 72 |  |  |
|  | 127183 | 127256 | - | trnD(GUC) |  | 74 |  |  |
|  | 127332 | 127419 | - | trnS(GCU) |  | 88 |  |  |
|  | 127496 | 127568 | + | trnM(CAU) |  | 73 |  |  |
|  |  |  |  |  | Total length | 107663 |  |  |
